# Influence of Public Assistance on Patients Receiving Extracorporeal Cardiopulmonary Resuscitation After Cardiac Arrest in Japan: A Retrospective Study

**DOI:** 10.7759/cureus.89194

**Published:** 2025-08-01

**Authors:** Takeshi Nishimura, Akihiko Inoue, Nana Hamamoto, Takuya Taira, Shinichi Ijuin, Toru Hifumi, Tetsuya Sakamoto, Yasuhiro Kuroda, Satoshi Ishihara

**Affiliations:** 1 Department of Emergency and Critical Care Medicine, Hyogo Emergency Medical Center, Kobe, JPN; 2 Department of Emergency and Critical Care Medicine, St. Luke's International Hospital, Tokyo, JPN; 3 Department of Trauma and Critical Care Center, Teikyo University School of Medicine, Tokyo, JPN; 4 Department of Emergency, Disaster and Critical Care Medicine, Kagawa University Hospital, Kagawa, JPN

**Keywords:** ecpr, low socioeconomic status, ohca, public assistance, ses

## Abstract

Objective

This study aimed to evaluate the influence of public assistance on patients with out-of-hospital cardiac arrest (OHCA) who received extracorporeal cardiopulmonary resuscitation (ECPR) in Japan.

Methods

We conducted a secondary analysis of data from the SAVE-J II study, a retrospective, multicenter registry study involving 36 participating institutions in Japan. Patients with cardiac arrest who received ECPR were divided into two groups, depending on whether or not they had received public assistance. The primary outcome was 30-day survival. Secondary outcomes were as follows: 30-day favorable neurological outcomes (Cerebral Performance Category scores 1-2); survival at discharge; favorable neurological outcome at discharge; number of Intensive Care Unit (ICU), hospital, ventilator, and extracorporeal membrane oxygenation (ECMO) days; medical expenses; proportion of percutaneous coronary intervention (PCI); target temperature management (TTM); mechanical circulatory support (MCS) device use; and withdrawal of life-sustaining therapy (WLST).

Results

Of the 2,157 patients registered in the SAVE-J II study, 1,885 were enrolled in this study; 99 patients (5.3%) received public assistance, and 1,786 patients (94.7%) did not. Multivariable logistic regression analysis did not show a significant difference in 30-day survival (OR: 1.22; 95% CI: 0.77-1.95; p = 0.40). Except for the use of MCS devices, there were no significant differences in secondary outcomes.

Conclusion

The use of public assistance was not associated with clinical outcomes or treatment options, except for MCS devices, among OHCA patients receiving ECPR. These results may imply that clinicians do not need to hesitate in implementing ECPR for OHCA patients receiving public assistance. Further studies on the association between socioeconomic status and ECPR are warranted.

## Introduction

Prognoses after cardiac arrest are often considered poor, and salvaging patients with out-of-hospital cardiac arrest (OHCA) remains challenging [1]. Recently, extracorporeal cardiopulmonary resuscitation (ECPR) - the deployment of veno-arterial extracorporeal membrane oxygenation (ECMO) to provide immediate cardiovascular support for patients with cardiac arrest - has been implemented to improve patient outcomes [2]. ECPR is recommended in settings where it can be implemented and may be regarded as a rescue therapy for selected cardiac arrest patients when conventional cardiopulmonary resuscitation (CPR) fails [3]. However, compared to conventional therapy, ECPR is expensive, which could be one reason why it is not widely used around the world. Additionally, there is little evidence supporting the cost-effectiveness of ECPR for OHCA patients.

It is well known that patients with lower incomes are associated with shorter life expectancy [4], and mortality after cardiac arrest is also influenced by income inequality [5]. Whereas high-income patients typically have better access to healthcare, low-income patients tend to have multiple comorbidities, lower levels of education, and higher rates of unemployment, which may lead to hesitation in seeking medical attention [6]. Furthermore, lower-income patients frequently have coronary risk factors [7], which may elevate the risk of cardiac arrest. In Japan, there are several types of insurers, which can be roughly divided based on three types of insurance: employer-based health insurance, residence-based national health insurance, and health insurance for people aged 75 and over. Notably, low-income individuals can receive free medical care by receiving public assistance, which is a safety net for those who cannot meet their basic needs [8]. Public assistance is assessed based on individual and household needs, considering income, assets, and other resources. In September 2023, more than 2 million people (about 1.6% of the total population) received public assistance. This medical system has been implemented to narrow the gap in treatment options and prognoses caused by income inequality. However, little is known about the influence of receiving public assistance among patients with OHCA, especially those who received ECPR.

This study aimed to evaluate whether public assistance had an influence on the survival and neurological outcomes and medical resource utilization among patients who received ECPR after OHCA.

## Materials and methods

This was a post-hoc analysis of the SAVE-J II study, which was a retrospective, multicenter registry study of OHCA patients who were resuscitated with ECPR. This study involved 36 tertiary emergency medical facilities in Japan. The SAVE-J II study included consecutive OHCA patients aged 18 years or older who were resuscitated with ECPR. They were admitted to the participating institutions between January 1, 2013, and December 31, 2018. The inclusion criterion was cardiac arrest at the time ECMO was initiated. The exclusion criteria were as follows: (1) patients who had sustained a return of spontaneous circulation (ROSC) when ECMO was initiated, (2) patients withdrawn from ECMO after cannulation due to ROSC, (3) patients transferred from other hospitals, and (4) patients with missing data on public assistance and outcomes. The SAVE-J II study was registered in the Kagawa University Hospital Medical Information Network Clinical Trials Registry (approval number: 2018-110). Institutional Review Boards at Hyogo Emergency Medical Center (2023007) approved this study. The local committee waived the requirement for patient consent. 

We compared two groups: patients who had received public assistance (PA group) and those who had not (non-PA group). The primary outcome was 30-day survival. Secondary outcomes were 30-day favorable neurological outcomes (defined as Cerebral Performance Category scores of 1 or 2), favorable neurological outcome at discharge, survival at discharge, number of days in the Intensive Care Unit (ICU), in the hospital, on a ventilator, and on ECMO, medical expenses during hospitalization, proportion of percutaneous coronary intervention (PCI), target temperature management (TTM), use of mechanical circulatory support (MCS) devices, including intra-aortic balloon pump (IABP)/Impella, and withdrawal of life-sustaining therapy (WLST). In this study, WLST was defined as patients from two combined categories: those with withholding of life-sustaining treatment and those with withdrawal of life-sustaining treatment. Withholding life-sustaining treatment is commonly considered the establishment of treatment limits, with no escalation of use of devices or drugs; withdrawal of life-sustaining treatment in Japan is commonly considered the termination of device use, pharmacological intervention, and/or other therapies [9].

Continuous variables were described using the median and interquartile range. Categorical variables were described using percentages and compared using the chi-square test. Discrete variables were evaluated using the Mann-Whitney U test.

Using public assistance as an independent variable, univariable and multivariable logistic regression were performed for 30-day survival, neurological outcomes, survival at discharge, and favorable neurological outcomes at discharge. Multivariable logistic regression analysis was adjusted for known risk factors such as age, sex, witnessed cardiac arrest, presence of bystander CPR, electrocardiogram on scene or at hospital arrival (shockable rhythm or not), place of cardiac arrest (public setting or non-public setting), and estimated low-flow time (defined as the time from collapse to initiation of ECMO).

The number of ICU, hospital, ventilator, and ECMO days, as well as medical expenses during hospitalization ($1 USD = 150 yen), were compared between the PA group and the non-PA group using the Mann-Whitney U test.

Also, whether or not PCI, TTM (including 32-34 degrees or >34 degrees), use of MCS devices, and WLST were used as treatment options during hospitalization was evaluated in the same logistic regression models.

In addition, Kaplan-Meier survival curves and log-rank tests comparing the PA and non-PA groups were conducted and depicted. The results of logistic regression are presented using ORs and 95% CIs. Statistical analysis was performed using STATA/IC 15 (StataCorp, Lakeway, TX, USA). Missing data were neither replaced nor estimated. A p-value < 0.05 was considered significant in all analyses.

## Results

**Figure 1:**
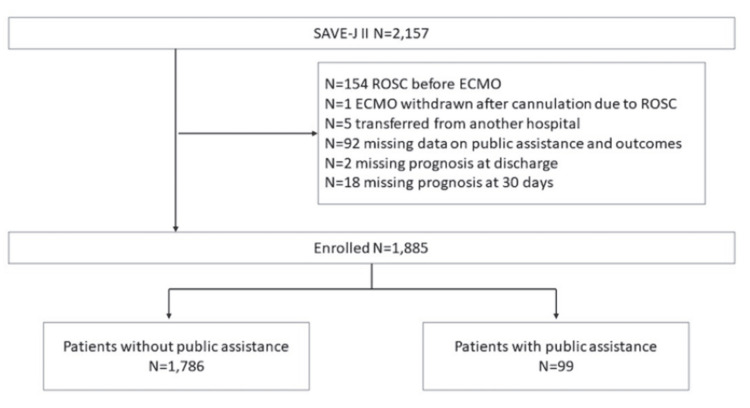
Flow chart depicting the inclusion of patients analyzed in this study. ROSC: return of spontaneous circulation; ECMO: extracorporeal membrane oxygenation

Among 2,157 available patients, 1,885 were enrolled in the study. Of those, 1,786 patients (94.7%) were in the non-PA group, and 99 patients (5.3%) were in the PA group (Figure 1).

Patient characteristics and backgrounds are summarized in Table 1. The proportion of patients with a spouse was lower in the PA group compared to the non-PA group (non-PA group: 60.1% (1,074/1,786) vs. PA group: 20.2% (20/99), p < 0.01). Medical history of cardiac disease was more frequent in the PA group (non-PA group: 6.2% (111/1,786) vs. PA group: 13.1% (13/99), p < 0.01), while hypertension was higher in the non-PA group (non-PA group: 31.0% (554/1,786) vs. PA group: 20.2% (20/99), p = 0.02). Witnessed cardiac arrest (non-PA group: 77.3% (1,380/1,786) vs. PA group: 64.6% (64/99), p < 0.01) and bystander CPR (non-PA group: 57.6% (1,029/1,786) vs. PA group: 43.4% (43/99), p < 0.01) occurred more frequently in the non-PA group. Notably, OHCA patients in the PA group were more often found on the street (non-PA group: 12.2% (218/1,786) vs. PA group: 18.2% (18/99)) and in public settings (non-PA group: 16.3% (291/1,786) vs. PA group: 21.2% (21/99)), whereas those in the non-PA group were more frequently found in the workplace (non-PA group: 11.4% (203/1,786) vs. PA group: 3.0% (3/99)).

**Table 1:** Characteristics and epidemiology of the enrolled patients. PA: public assistance; CPR: cardiopulmonary resuscitation; PEA: pulseless electrical activity; ROSC: return of spontaneous circulation; ECMO: extracorporeal membrane oxygenation

Variables	Total	Missing	Non-PA	PA	p-value
	n = 1,885		n = 1,786	n = 99	
Age, year	61 (49-69)	1	61 (49-69)	61 (53-69)	0.17
Sex, male	1,546 (82.0)	0	1,461 (81.8)	85 (85.9)	0.31
Spouse	1,085 (57.6)	132	1,065 (59.6)	20 (20.2)	<0.01
Performance status					0.98
0-1	1,801 (95.5)	-	1,710 (95.7)	91 (91.9)	-
2-4	39 (2.1)	-	37 (2.1)	2 (2.0)	-
Unknown	45	-	39	6	-
Medical history of coronary risk factors					
Hypertension	574 (30.5)	0	554 (31.0)	20 (20.2)	0.02
Diabetes mellitus	354 (18.8)	0	338 (18.9)	16 (16.2)	0.49
Cardiac disease	124 (6.6)	0	111 (6.2)	13 (13.1)	<0.01
Renal failure	89 (4.7)	0	84 (4.7)	5 (5.1)	0.87
Prehospital					
Witnessed cardiac arrest	1,444 (76.6)	7	1,380 (77.3)	64 (64.6)	<0.01
Bystander CPR	1,072 (56.9)	31	1,029 (57.6)	43 (43.4)	<0.01
Location of cardiac arrest					<0.01
Home	760 (40.3)	-	738 (41.3)	39 (39.4)	-
Public space	312 (16.6)	-	291 (16.3)	21 (21.2)	-
Street	236 (12.5)	-	218 (12.2)	18 (18.2)	-
Workplace	206 (10.9)	-	203 (11.4)	3 (3.0)	-
Others	371 (19.7)	-	336 (18.8)	18 (18.2)	-
Initial cardiac rhythm					0.06
Shockable rhythm	1,185 (62.9)	-	1,125 (63.0)	61 (60.4)	-
PEA	495 (26.3)	-	475 (26.6)	21 (20.8)	-
Asystole	186 (9.9)	-	170 (9.5)	16 (15.8)	-
Others	19 (1.0)	-	16 (0.9)	3 (3.0)	-
Transient ROSC before hospital arrival (yes/no)	179/1,676	30	171/1,585	8/91	0.59
On hospital					
Cardiac rhythm on arrival					0.83
Shockable rhythm	862 (45.7)	-	818 (45.8)	44 (44.4)	-
PEA	606 (32.1)	-	577 (32.3)	29 (29.3)	-
Asystole	393 (20.8)	-	369 (20.7)	24 (24.2)	-
Others	24 (1.3)	-	22 (1.2)	1 (1.0)	-
Mechanism of cardiac arrest					<0.01
Internal-heart	1,364 (72.4)	-	1,301 (72.8)	63 (63.6)	-
Internal-non heart	166 (8.8)	-	156 (8.7)	10 (10.1)	-
External	101 (5.4)	-	85 (4.8)	16 (16.2)	-
Unknown	254 (13.5)	-	242 (13.5)	10 (10.1)	-
Targeted temperature management (yes/no)	1,246/639	0	1,175/611	71/28	0.23
Time course					
Onset-hospital	34 (26-43)	236	34 (26-43)	34 (27-43)	0.98
Hospital arrival-ECMO establishment	23 (16-34)	73	23 (16-34)	22 (16-31)	0.59
Estimated low flow time	59 (47-73)	102	59 (47-73)	56.5 (47-64)	0.19

Multivariable logistic regression analysis did not reveal a difference in 30-day survival between the two groups (non-PA group: 25.8% (461/1,786) vs. PA group: 28.3% (28/99), OR: 1.22, 95% CI: 0.77-1.95, p = 0.40) (Table 2). Additionally, there were no significant differences in secondary outcomes, including 30-day favorable neurological outcomes (non-PA group: 13.7% (244/1,786) vs. PA group: 16.2% (16/99), OR: 1.46, 95% CI: 0.82-2.58, p = 0.20), survival at discharge (non-PA group: 25.6% (457/1,786) vs. PA group: 29.3% (29/99), OR: 1.31, 95% CI: 0.82-2.08, p = 0.26), and favorable neurological outcomes at discharge (non-PA group: 13.8% (247/1,786) vs. PA group: 17.2% (17/99), OR: 1.59, 95% CI: 0.91-2.77, p = 0.11).

**Table 2:** Multivariable logistic regression analysis of secondary outcomes. Regression analysis revealed that 30-day survival, 30-day favorable neurological outcome, survival at discharge, and favorable outcome at discharge were not associated with public assistance use. *Adjusted for the use of public assistance, age, sex, witnessed cardiac arrest, bystander cardiopulmonary resuscitation, electrocardiogram rhythm (shockable or not), site (public or not), and estimated low flow time. CPC: cerebral performance category

Variables	Non-PA	PA	Unadjusted OR (95% CI)	p-value	Adjusted OR (95% CI)*	p-value
	n = 1,786	n = 99				
30-day survival	461 (25.8)	28 (28.3)	1.13 (0.72-1.78)	0.59	1.22 (0.77-1.95)	0.4
30-day favorable neurological outcome	244 (13.7)	16 (16.2)	1.22 (0.70-2.12)	0.48	1.46 (0.82-2.58)	0.2
Survival at discharge	457 (25.6)	29 (29.3)	1.20 (0.77-1.88)	0.41	1.31 (0.82-2.08)	0.26
Favorable CPC score at discharge	247 (13.8)	17 (17.2)	1.29 (0.75-2.21)	0.35	1.59 (0.91-2.77)	0.11

The number of ICU days (non-PA group: 3 (1-10) vs. PA group: 3 (1-9), p = 0.60), hospital days (non-PA group: 3 (1-18) vs. PA group: 4 (1-18), p = 0.48), ventilator days (non-PA group: 3 (1-8) vs. PA group: 3 (1-10), p = 0.25), and ECMO days (non-PA group: 3 (2-5) vs. PA group: 3 (2-4), p = 0.34) did not differ significantly (Table 3). Medical expenses (non-PA group: $14,600 ($6,600-$24,500) vs. PA group: $14,300 ($6,300-$25,500), p = 0.80) were also similar.

**Table 3:** Number of Intensive Care Unit days, hospital days, ventilator days, extracorporeal membrane oxygenation days, and medical expenses during hospitalization compared between the PA and non-PA groups. No significant differences were detected between the two groups. ECMO: extracorporeal membrane oxygenation; PA: public assistance

Variables	Non-PA	PA	p-value
	n = 1,786	n = 99	
Number of ICU days	3 (1-10)	3 (1-9)	0.6
Number of hospital days	3 (1-18)	4 (1-18)	0.48
Number of ventilator days	3 (1-8)	3 (1-10)	0.25
Number of ECMO days	3 (2-5)	3 (2-4)	0.34
Medical expenses ($)	14,600 (6,600-24,500)	14,300 (6,300-25,500)	0.8

Treatment options, including PCI (non-PA group: 40.4% (701/1,736) vs. PA group: 33.3% (31/93), OR: 0.70, 95% CI: 0.44-1.11, p = 0.13), TTM (non-PA group: 65.8% (1,175/1,786) vs. PA group: 71.7% (71/99), OR: 1.32, 95% CI: 0.83-2.11, p = 0.24), and WLST (non-PA group: 27.5% (492/1,786) vs. PA group: 25.3% (25/99), OR: 0.91, 95% CI: 0.57-1.45, p = 0.68), did not differ (Table 4). However, the use of MCS devices (non-PA group: 57.4% (1,001/1,743) vs. PA group: 45.8% (44/96), OR: 0.63, 95% CI: 0.41-0.98, p = 0.04) was lower in the PA group.

**Table 4:** Association between treatment options and public assistance. Treatment options, including PCI, targeted temperature management, and WLST, did not differ between the PA group and the non-PA group. However, the use of MCS devices (IABP/Impella) was lower in the PA group. *Adjusted for the use of public assistance, age, sex, witnessed cardiac arrest, bystander CPR, electrocardiogram rhythm (shockable or not), site of cardiac arrest (public or not), and estimated low-flow time. PA: public assistance; IABP: intra-aortic balloon pump; PCI: percutaneous coronary intervention; TTM: targeted temperature management; MCS: mechanical circulatory support; WLST: withholding/withdrawal of life-sustaining therapy

Variables	Non-PA	PA	Unadjusted OR (95% CI)	p-value	Adjusted OR (95% CI)*	p-value
	n = 1,786	n = 99				
PCI (%)	701/1,736 (40.4)	31/93 (33.3)	0.74 (0.47-1.15)	0.18	0.70 (0.44-1.11)	0.13
TTM (%)	1,175/1,786 (65.8)	71/99 (71.7)	1.32 (0.84-2.06)	0.23	1.32 (0.83-2.11)	0.24
32-34°C (%)	752 (64.0)	46 (64.8)	1.04 (0.63-1.71)	0.89	1.09 (0.65-1.08)	0.75
>34°C (%)	423 (36.0)	25 (35.2)	Reference	-	Reference	-
MCS devices (%)	1,001/1,743 (57.4)	44/96 (45.8)	0.63 (0.42-0.95)	0.03	0.63 (0.41-0.98)	0.04
WLST (%)	492/1,786 (27.5)	25/99 (25.3)	0.62 (0.56-1.41)	0.62	0.91 (0.57-1.45)	0.68

**Figure 2:**
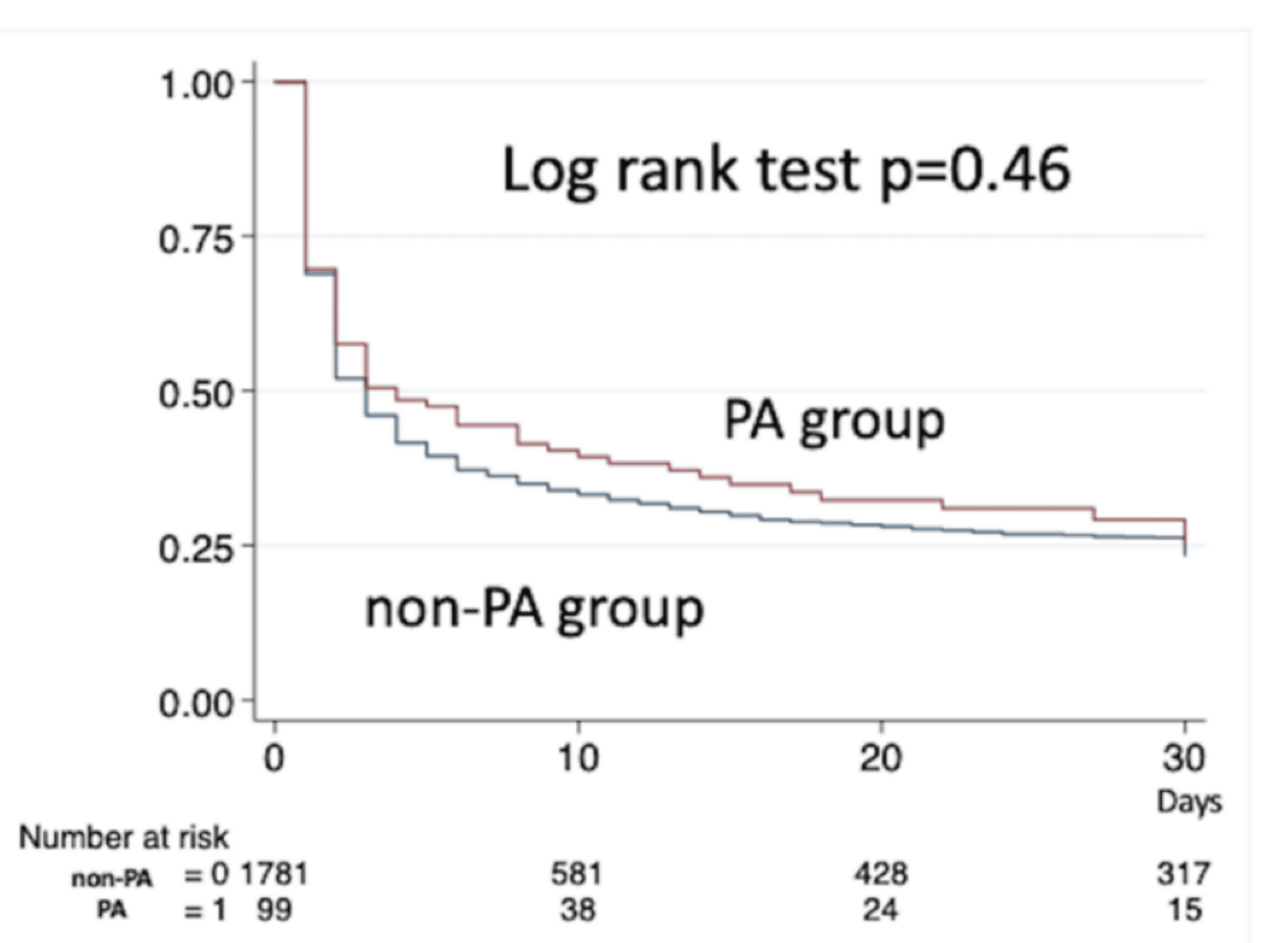
Kaplan-Meier survival curve between the PA group and the non-PA group within 30 hospital days. PA: public assistance

Kaplan-Meier curves within 30 days, using the log-rank test, were presented (Figure 2). The use of public assistance was not associated with improved survival outcomes (p = 0.46).

## Discussion

This study aimed to examine whether the use of public assistance influences the prognosis and treatment options among OHCA patients who received ECPR in Japan. Logistic regression analysis did not demonstrate that public assistance was associated with 30-day survival, and the Kaplan-Meier curve supported this result. There were no significant differences in clinical outcomes and treatment options utilized during hospitalization, including medical expenses, between the two groups, except for the use of MCS devices. This study found no objective reason for clinicians to hesitate in implementing ECPR for OHCA patients receiving public assistance.

Generally, it takes a few days after hospital admission for the patient’s type of insurance to be recognized. In the PA group, five cases had IABP reinsertion during hospitalization. Presumably, the use of MCS devices could be influenced by decisions made by the patients' families and clinicians if the patient was unconscious. We assumed that social isolation might be associated with the lower rate of use of these devices in the PA group. Indeed, the proportion of patients with a spouse was lower in the PA group. From the perspective of medical professionals, clinicians might emotionally hesitate to perform additional advanced treatments, such as Impella, for OHCA patients in the PA group. Although Impella was not commonly used during the study period, this novel advanced MCS device has become increasingly prevalent in recent years. The results may differ if the same analysis is conducted today. Further investigation is warranted.

Although similar studies on the association between income inequality and OHCA have been reported [5], results from these studies differed depending on the study location, since different countries/areas have different medical insurance systems. In Japan, universal coverage charges patients a very low cost, covering most hospitalization costs under national health care expenditures [10]. If support is insufficient for their living expenses, low-income people can apply for the public assistance system, including free medical care. Of note, it was demonstrated that lower household income and receiving public assistance were associated with higher mortality among COVID-19 patients [11]. In Taiwan and France, which have medical systems similar to that in Japan, low-income patients do not have to pay for medical care when receiving public assistance, and among those patients, those with acute myocardial infarction had poor prognoses [12]. The use of public assistance and the universal healthcare system in Japan still warrants discussion and raises concerns about issues in the future.

When it is difficult to accurately measure patient income directly, socioeconomic status could be a reliable surrogate, since income and socioeconomic status are strongly associated with each other. Also, the influence of socioeconomic status on cardiovascular outcomes is well known [13]. Although little is known about how much public assistance influences prognoses among OHCA patients, socioeconomic disparities play a pivotal role in the improved survival rate among OHCA patients [14,15]. Risk factors for cardiac arrest are strongly affected by socioeconomic status. Patients with lower socioeconomic status, as measured by education, income, and occupation, have a higher prevalence of diabetes and cardiovascular disease [7]. Poor education is related to insufficient management of lifestyle-related diseases [16]. Additionally, automated external defibrillator use is associated with socioeconomic disparities, educational level, and occupational inequalities [17-19]. Meanwhile, patients with public assistance tend to smoke and have poor control of coronary risk factors [20]. Since socioeconomic status, income, and public assistance are strongly correlated with each other, we used receipt of public assistance as a surrogate for low-income status in this study. However, our findings regarding previous medical history were not consistent with those from previous reports, and no direct relationship between public assistance and cardiac arrest was found, which may imply that patients on public assistance in Japan do not always represent those of low income and lower socioeconomic status. Unfortunately, our study design did not reflect the influence of educational level and occupation because of the lack of these data. A comprehensive study design that includes several confounders would be desirable for future research.

Even after ROSC, post-OHCA survival has been found to be consistently associated with income inequality. Income disparities are likely to be associated with physical resilience, which is influenced by the level of comorbidities [21]. In addition, a wide variation in post-arrest care was found, even within the same territories, and may affect the rate of survival to discharge [22], which means community-level socioeconomic status, where cardiac arrest occurs, may influence outcomes [23]. Although a study on patients in North American regions showed that post-cardiac arrest care, including TTM and PCI, might be associated with improved neurologic recovery [24], patients with government/Medicare insurance in the US state of California were found to be unlikely to have received cardiac catheterization [25]. Furthermore, lower income, education, and occupation were associated with a lower chance of receiving TTM and PCI [26]. Short-term and long-term outcomes after OHCA were associated with socioeconomic status, presumably due to the wide range of hospital care [27]. Compared to these previous reports, our study indicates that Japan’s medical system, supported by public assistance, worked appropriately and did not result in significant differences in outcomes and post-cardiac arrest care for lower-income OHCA patients who received ECPR. 

Although the chance of receiving treatment options and prognoses in the current study did not differ between the two groups, the cost-effectiveness of ECPR in the PA group is a concerning issue, and health-related quality of life after cardiac arrest should be assessed [28]. The cost-effectiveness of ECPR, evaluated using quality-adjusted life years, was found to be comparable to that of patients who received heart transplants [29].

Additionally, cost-utility among consecutive ECPR patients in Australia was below the willingness-to-pay threshold but economically acceptable [30]. A multicenter, retrospective analysis in Japan demonstrated that the cost-effectiveness of ECPR was acceptable compared to that of conventional resuscitative strategies [31]. However, all these studies were retrospective and had subjective analyses; thus, loss to follow-up was frequently recognized due to the nature of observational studies [32]. No studies about the association between public assistance and the cost-effectiveness of ECPR, including long-term prognoses, have been reported. 

Limitations

This study had some limitations. First, although the data were obtained from the ECPR registry in Japan, no protocols for each treatment were established, and the decision to implement ECPR was made at each physician’s discretion and according to each institution’s policy. This may have caused selection bias between the two groups. Second, the hospitals implementing ECPR were generally located in urban areas, which may have caused selection bias. Indeed, 5.3% of patients were receiving public assistance in our study, which was a higher proportion compared to that of actual public assistance recipients (about 1.6% of the total population). Third, we used public assistance as a surrogate variable for disparities in income and socioeconomic status; therefore, patients’ individual incomes might not be reflected by public assistance. Fourth, the type of insurance occasionally could not be found until a few days after hospital admission. Thus, in the Emergency Department, treatment options were not always influenced by whether the patients used public assistance or not. To examine detailed information during hospitalization, we evaluated several outcomes during hospitalization, which did not reveal large differences. Lastly, long-term prognoses after hospital discharge were not evaluated because these data were not available in the original database. 

## Conclusions

Our study demonstrated that the use of public assistance was not associated with clinical outcomes and treatment options, except for MCS devices, among OHCA patients receiving ECPR. These results may imply that clinicians don’t have to hesitate about implementing ECPR for OHCA patients with the use of public assistance. Further studies on the association between socioeconomic status and ECPR are warranted.
